# A novel seven-long non-coding RNA signature predicts survival in early stage lung adenocarcinoma

**DOI:** 10.18632/oncotarget.14781

**Published:** 2017-01-21

**Authors:** Mingwei Chen, Baoquan Liu, Jianbing Xiao, Yingnan Yang, Yafang Zhang

**Affiliations:** ^1^ Department of Anatomy, Harbin Medical University, Harbin 150081, PR China; ^2^ Department of Thoracic Surgery, The Third Affiliated Hospital of Harbin Medical University, Harbin 150040, PR China

**Keywords:** long non-coding RNAs, lung adenocarcinoma, survival, prognosis

## Abstract

Increasing evidence has revealed the significant association between dysregulated lncRNA expression and cancers. The prognostic value of lncRNAs in predicting the risk of disease recurrence and identifying high-risk subgroup of early stage lung adenocarcinoma (LUAD) is still unclear. In this study, we analyzed lncRNA expression profiles of 415 early-stage LUAD patients from Gene Expression Omnibus and identified a novel seven-lncRNA signature that was significantly associated with survival in patients with early-stage LUAD (HR = 2.718, CI = 2.054–3.597, *p* < 0.001). Based on the seven-lncRNA signature, we constructed a risk score model which is able to classify patients of training dataset into the high-risk group and the low-risk group with significantly different clinical outcome (*p* < 0.001). The robustness of the seven-lncRNA signature was successfully validated through application in other two independent patient datasets. Furthermore, the prognostic value of seven-lncRNA signature was independent of other clinicopathological factors including age, gender, stage and smoking status. Functional analysis suggested that the seven-lncRNA signature may be involved in a variety of biological pathways including cell cycle, ECM-receptor interaction, Focal adhesion and p53 signaling pathway. Taken together, our study not only provides insights into the lncRNA association with LUAD, but also provide alternative molecular markers in prognosis prediction for early-stage LUAD patients.

## INTRODUCTION

Lung cancer is the most common cancer in both men and women for several decades and is still the leading cause of cancer death worldwide including China [1]. In China, it is estimated that there were 733,000 newly cases of lung cancer and more than 610,000 deaths in 2015, which ranked in the top 1 of common cancers for men and top 2 for women [2, 3]. Lung cancer comprised of two main types: non-small-cell lung cancer (NSCLC) accounting for approximately 85% and small-cell lung cancers. Lung adenocarcinoma (LUAD) is one of three histological subtypes of NSCLC. The incidence of LUAD has increased markedly and become the most predominant types of NSCLC, which constitute nearly 50% of NSCLC cases in China [4]. Surgical resection is currently the treatment standard for LUAD patients with early stage. After surgical resection, patients with stage IB or stage II often receive additional adjuvant chemotherapy to improve their survival rate by 5% to 10% [5]. However, 40% for stage IB and 66% of stage II patients still faced relapse and will die as a result of disease recurrence [6, 7]. On the other hand, patients with completely resected stage IA are not recommended for considering, but still have a relapse rate as high as 30% [7]. Therefore, besides traditional clinical factors, it is urgently need to develop novel molecular prognostic signature for predicting the risk of disease recurrence and identifying high-risk subgroup of early stage LUAD patients who might benefit from adjuvant treatment

Increasing studies in the human genome and transcriptome have suggested that only ~2% of the human genome sequence encodes only ~20,000 protein-coding genes, whereas most of the rest were transcribed into RNA transcripts with no or little protein coding capacity [8]. Non-coding RNAs (ncRNAs) were comprised of two major classes based on their size: small ncRNAs and long non-coding RNAs (lncRNAs). LncRNAs are generally defined as ncRNAs ranging in length from 200 nt to ~100 kilobases (kb) and are frequently transcribed by RNA polymerase II [9]. More and more evidence has shown that lncRNAs play critical regulatory roles in many biological and pathological processes [10–13]. Transcriptional profiling analysis has revealed highly altered lncRNA expression patterns in cancer tissues compared to normal tissues [14]. Many known lncRNAs have been observed as having oncogenic and tumor suppressive roles during cancer progression (such as *MEG3*, *MALAT1* and *HOTAIR*), demonstrated potential applications of lncRNAs in clinical diagnosis, prognosis and treatment like mRNAs and microRNAs (miRNAs) [15, 16]. Recent studies have provided evidence supporting lncRNAs as useful molecular markers in diagnosis and prognosis prediction, and several novel expression-based lncRNA signature were identified in multiple human cancers [17–23]. Recently, some efforts have been undertaken to identify lncRNA-based signature for predicting survival in NSCLC [24, 25]. However, the prognostic value of lncRNAs in predicting the risk of disease recurrence and identifying high-risk subgroup of early stage LUAD patients is still unclear.

In this study, to construct a reliable prognostic lncRNA signature that could identify early-stage LUAD patients with a high risk of disease recurrence, we analyzed lncRNA expression profiles of 415 early-stage LUAD patients from Gene Expression Omnibus and developed a prognostic seven-lncRNA signature to predict survival.

## RESULTS

### Identification of lncRNA biomarkers significantly associated with survival from the training dataset

The GSE50081 dataset derived from GEO database was used as the training dataset for identifying prognostic lncRNAs in early stage LUAD patients. By subjecting expression data of 2332 lncRNAs in 127 patients from the training dataset to the univariate Cox regression model, a total of 48 lncRNAs were identified as candidate biomarkers significantly associated with survival from the training dataset (adjusted *p*-value < 0.05 after Bonferroni correction) (Supplementary Table 1). To take into account for the interrelated relationship among 48 lncRNAs, we performed multivariate Cox regression analysis for 48 lncRNAs and identified seven lncRNAs as independent biomarkers predicting survival in LUAD patients (*p* < 0.05) (Table 1). Among them, five lncRNAs have positive coefficient indicating that their high expression was associated with poor survival, whereas the remaining two lncRNAs have negative coefficient indicating that their high expression was associated with better survival.

**Table 1:** LncRNAs significantly associated with the survival in the training dataset

Ensembl id	Gene name	Genomic location	Hazard ratio	Coefficient	Adjusted *p*-value
ENSG00000280278.1	FLJ30679	Chr 16: 86,555,320–86,557,299 (+)	1.621	0.483	0.032
ENSG00000227036.6	LINC00511	Chr 17: 72,323,123–72,640,472 (−)	1.571	0.452	0.042
ENSG00000269427.1	CTC-429P9.1	Chr 19: 16,630,743–16,643,942 (+)	0.57	−0.562	0.031
ENSG00000281162.2	LINC01127	Chr 2: 101,962,056–101,987,167 (+)	1.959	0.672	0.022
ENSG00000218537.1	MIF-AS1	Chr 22: 23,894,426–23,898,930 (−)	1.557	0.443	0.042
ENSG00000279130.1	RP11-278J6.4	Chr 5: 143,406,959–143,407,420 (+)	1.598	0.469	0.042
ENSG00000167912.5	RP11-25K19.1	Chr 8: 59,119,040–59,121,346 (+)	0.514	−0.665	0.014

### Derivation of a seven-lncRNA signature in predicting survival for early stage LUAD patients

**Figure 1:**
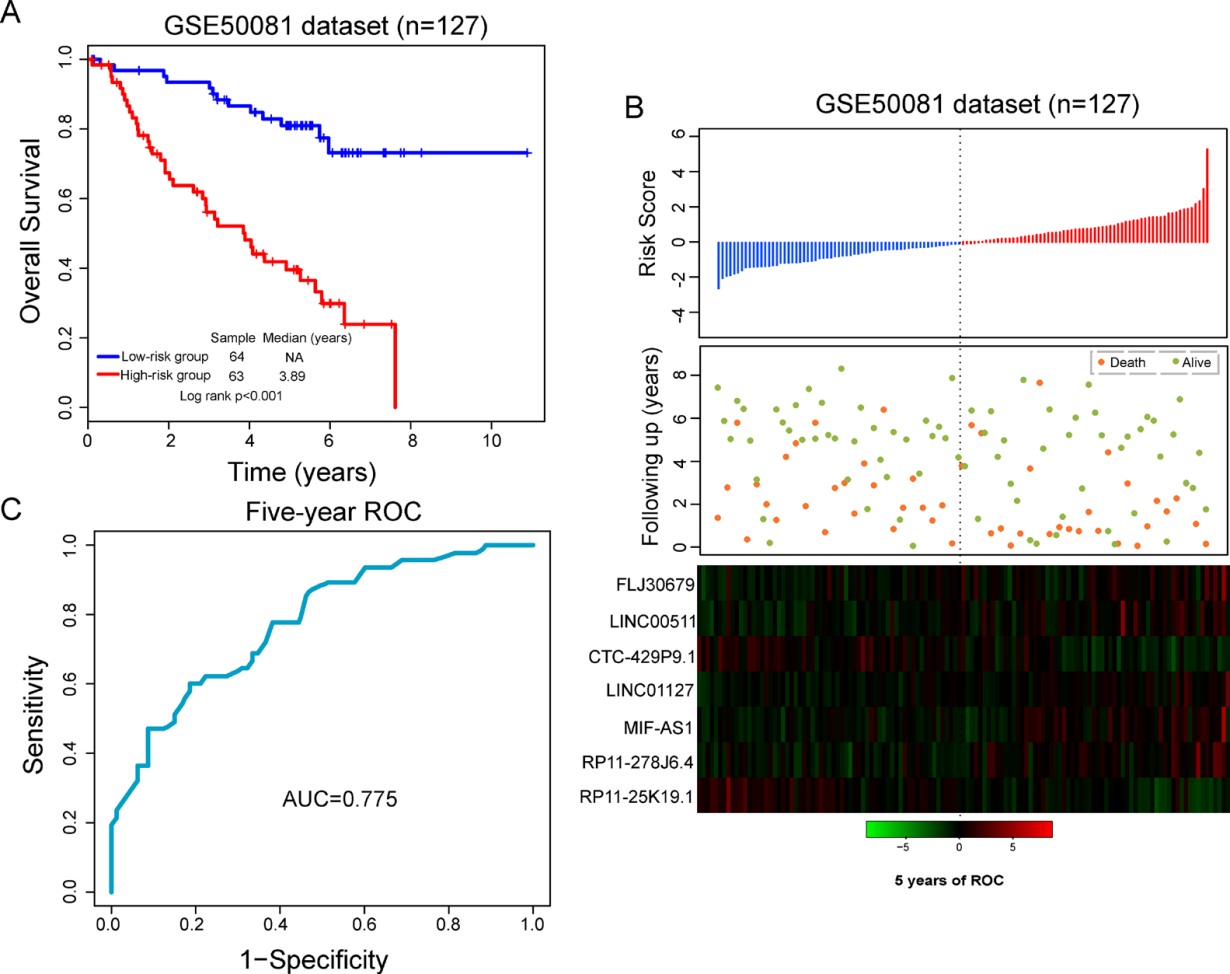
Performance evaluation of the seven-lncRNA signature in the training dataset (**A**) Kaplan-Meier survival curves between high-risk group and low-risk group. (**B**) The distribution of risk score, patients’ survival status and expression patterns of lncRNA signature. (**C**) Five-year ROC curves of the seven-lncRNA signature.

In order to build a clinically available risk prediction model, these seven independent biomarkers were integrated into a seven-lncRNA signature by risk scoring method as previously described [18, 25, 26]. Firstly, seven lncRNA biomarkers were subjected to multivariate Cox regression model to obtain their relative contribution power for predicting survival. Then seven-lncRNA signature-based risk score model was constructed as described in the Methods section. Using above risk score model, each patient of the training dataset was assigned a risk score according to expression value of seven lncRNA biomarkers, and then was classified as high-risk or low-risk patient using the median risk score as the cutoff value. As a result, patients of the training dataset were divided into the high-risk group (*n* = 63) and low-risk group (*n* = 64) with significantly different survival (*p* < 0.001) (Figure 1A). Moreover, the seven-lncRNA signature was significantly associated with survival of early stage LUAD patients (HR = 2.718, 95% CI = 2.054–3.597, *p* < 0.001) (Table 2). The median survival of patients with high-risk scores was 3.69 years, which is significantly lower than those of low-risk patients with not reach median survival (*p* < 0.001) (Figure 1A).

**Table 2:** Univariate cox regression analysis for different patient datasets in this study

Variable	Unfavorable/Favorable	Hazard Ratio	95% CI	*p*-value
GSE50081 dataset				
Seven-lncRNA signature	High-risk/Low-risk	2.718	2.054–3.597	< 0.001
Age	> 65/<=65	1.455	0.774–2.735	0.244
Gender	Male/Female	1.410	0.807–2.463	0.228
Stage	II/I	2.443	1.383–4.316	0.002
Smoking	Yes/No	1.662	0.742–3.723	0.217
GSE21210 dataset				
Seven-lncRNA signature	High-risk/Low-risk	1.579	1.151–2.168	0.005
Age	> 65/<=65	2.779	1.349–5.724	0.006
Gender	Male/Female	1.686	0.818–3.476	0.157
Stage	II/I	4.297	2.092–8.828	< 0.001
Smoking	Yes/No	0.524	0.252–1.089	0.084
GSE30219 dataset				
Seven-lncRNA signature	High-risk/Low-risk	1.467	1.131–1.903	0.004
Age	> 65/<=65	1.816	0.999–3.3	0.050
Gender	Male/Female	1.124	0.541–2.336	0.754
Stage	II/I	2.117	1.08–4.151	0.029

The distribution of risk score, survival status of LUAD patients and expression patterns of the seven-lncRNA signature was shown in Figure 1B. As shown in Figure 1B, patients with high-risk scores tended to express five lncRNAs with a positive coefficient, whereas patients with low-risk scores tended to express two lncRNAs with a negative coefficient. The five-year ROC curve of seven-lncRNA signature achieved an AUC of 0.775 (Figure 1C). The survival rates of patients in the high-risk group were 56.1% and 39.6% at three and five years, respectively, whereas the corresponding rates in the low-risk group were 91.8% and 81%. These results indicated that the seven-lncRNA signature was able to distinguish LUAD patients with high or low risk of survival.

### Validation of the seven-lncRNA signature in the independent patient dataset

**Figure 2:**
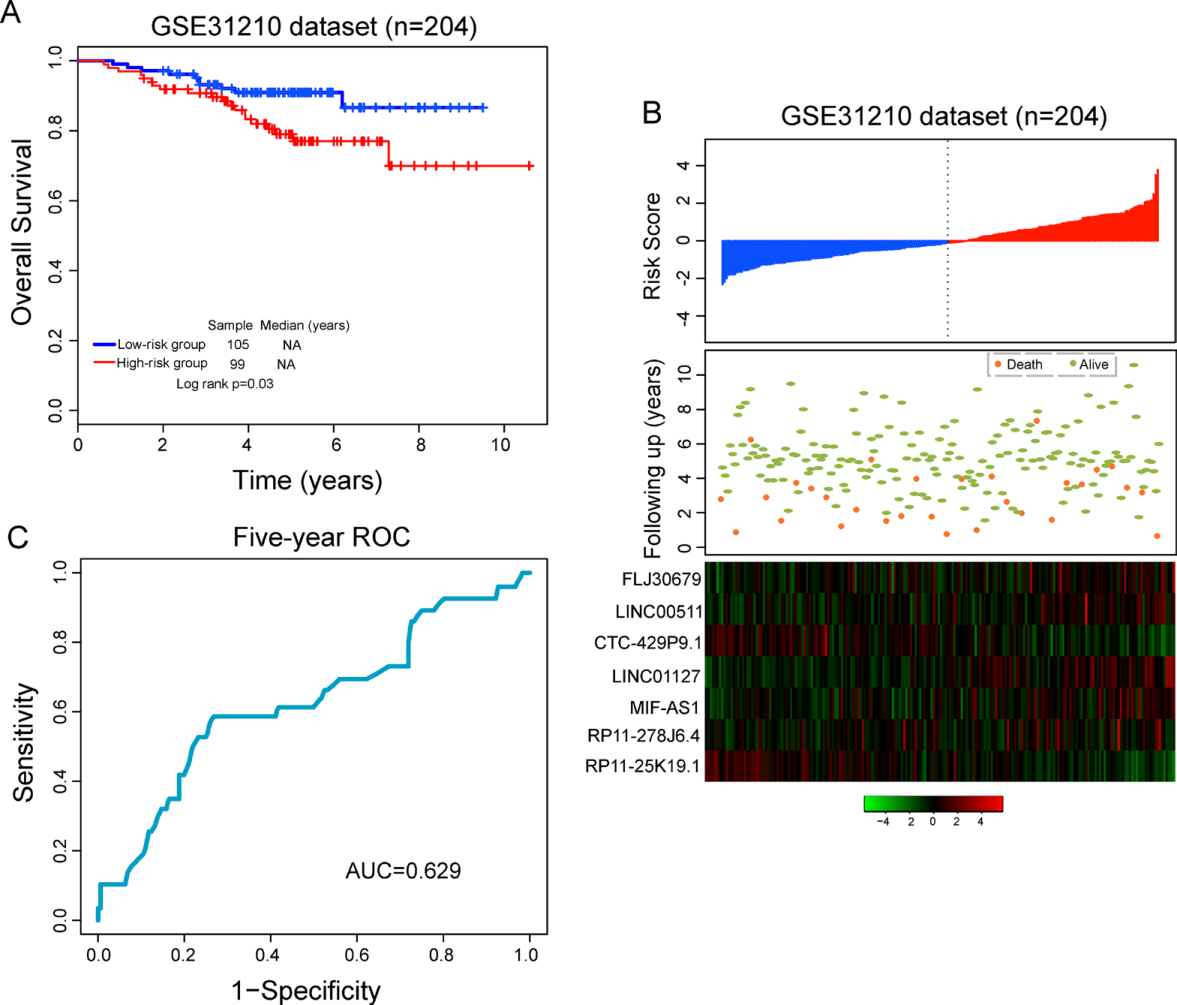
Validation of predictive value of the seven-lncRNA signature in the independent GSE31210 dataset (**A**) Kaplan-Meier survival curves between high-risk group and low-risk group. (**B**) The distribution of risk score, patients’ survival status and expression patterns of lncRNA signature. (**C**) Five-year ROC curves of the seven-lncRNA signature.

To evaluate the reproducibility of the seven-lncRNA signature, we validated its predictive ability using an independent LUAD dataset of 204 patients from GEO database (accession is GSE31210). The seven-lncRNA signature risk score for each of 204 patients in GSE31210 dataset was calculated using the same risk score model from the training dataset without changing parameters. By using the same cutoff value derived from the training dataset, patients in the GSE31210 dataset were then classified into the high-risk group (*n* = 99) and low-risk group (*n* = 105). Similar to the findings from the training set, survival analysis demonstrated significantly different survival between predicted two groups (*p* = 0.03, Figure 2A). Patients with high-risk score tended to have poor survival than that of patients with the low-risk score. Univariate analysis suggested that there is a significant association between risk score and survival of LUAD patients (HR = 1.579, 95% CI = 1.151–2.168, *p* = 0.005) (Table 2)

The distribution of risk score, survival status of LUAD patients and expression patterns of the seven-lncRNA signature was shown in Figure 2B. As shown in Figure 2B, patients with high-risk scores tended to express five lncRNAs with a positive coefficient, whereas patients with low-risk scores tended to express two lncRNAs with a negative coefficient. The five-year ROC curve of seven-lncRNA signature achieved an AUC of 0.629 (Figure 2C). The three-year and five-year survival rates of the high-risk group were 89.6% and 77%, respectively, whereas the corresponding rates in the low-risk group were 93.1% and 91%, respectively. These results with GSE31210 dataset indicated that the seven-lncRNA signature was robust to identify patients with poor survival for early stage LUAD.

### Further validation of the seven-lncRNA signature with another independent dataset

**Figure 3:**
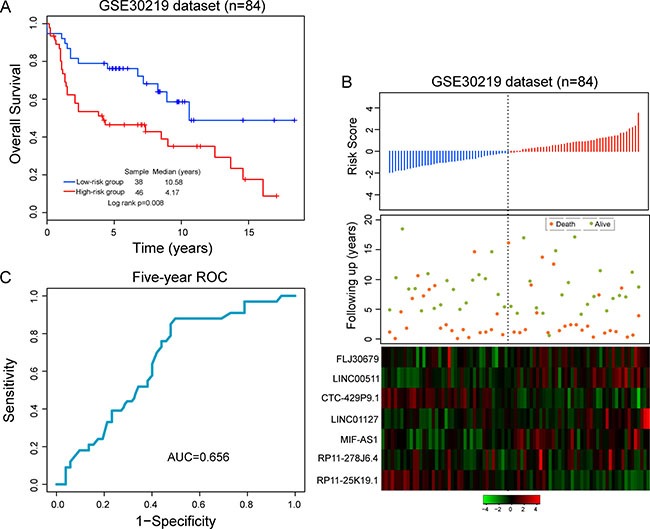
Further confirmation of predictive value of the seven-lncRNA signature in another independent GSE30219 dataset (**A**) Kaplan-Meier survival curves between high-risk group and low-risk group. (**B**) The distribution of risk score, patients’ survival status and expression patterns of lncRNA signature. (**C**) Five-year ROC curves of the seven-lncRNA signature.

To further testing the robustness of the seven-lncRNA signature in early stage LUAD patients, we validated the predictive power of the seven-lncRNA signature in another independent LUAD dataset of 84 patients from GEO database (accession is GSE30219). All patients were divided into the high-risk group (*n* = 46) and low-risk group (*n* = 38) according to the risk score model and cutoff value derived from the training dataset. As in the training and GSE31210 datasets, the predicted two groups of patients revealed significantly different survival. Patients with high-risk scores had significantly shorter survival than those with low-risk scores (median survival 4.17 years vs. 10.58 years, *p* = 0.008) (Figure 3A). In univariate analysis, the HR of patients with high-risk score vs. those with low risk score for survival were 1.467 (*p* = 0.004; 95% CI = 1.131–1.903).

The distribution of risk score, survival status of LUAD patients and expression patterns of the seven-lncRNA signature was shown in Figure 3B. As shown in Figure 3B, patients with high-risk score tended to express five risky lncRNAs and patients with low-risk score tended to express two protective lncRNAs. The AUC for the seven-lncRNA signature was 0.656 at five-year of survival (Figure 3C). In the high-risk group, the survival rates of patients were 53.4% and 46.6% at three and five years, respectively, which were also significantly lower than those in the low-risk group whose corresponding proportions were 78.9% and 76.2%.

### The prognostic value of seven-lncRNA signature is independent of other clinicopathological factors

To examine whether the prognostic value of the seven-lncRNA signature is independent of other clinical variables, we conducted multivariate Cox proportional hazard regression analysis with risk score and other available clinicopathological factors (including age, gender, stage and smoking status) as covariates in three LUAD patient datasets. Multivariate regression analysis showed that the seven-lncRNA signature still was significantly associated with survival when adjusted for age, gender, stage and smoking status in the training dataset (HR = 2.699, 95% CI = 1.985–3.669, *p* < 0.001) and in the other two independent patient datasets (HR = 1.391, 95% CI = 1.002–1.972, *p* = 0.047 for GSE21210 and HR = 1.589, 95% CI = 1.184–2.132, *p* = 0.002 for GSE30219) (Table 3). However, we also found that besides seven-lncRNAs signature, age and stage were significant in the multivariate analysis in some of three datasets.

**Table 3:** Multivariate cox regression analysis for different patient cohorts in this study

Variable	Unfavorable/Favorable	Hazard Ratio	95% CI	*p*–value
GSE50081 dataset				
Seven–lncRNA signature	High–risk/Low–risk	2.699	1.985–3.669	< 0.001
Age	> 65/<=65	1.386	0.727–2.639	0.321
Gender	Male/Female	1.443	0.797–2.614	0.226
Stage	II/I	1.595	0.862–2.951	0.137
Smoking	Yes/No	0.719	0.297–1.736	0.503
GSE21210 dataset				
Seven–lncRNA signature	High–risk/Low–risk	1.391	1.002–1.972	0.047
Age	> 65/<=65	3.558	1.694–7.473	0.001
Gender	Male/Female	0.993	0.356–2.766	0.989
Stage	II/I	3.875	1.842–8.152	< 0.001
Smoking	Yes/No	0.570	0.204–1.591	0.283
GSE30219 dataset				
Seven–lncRNA signature	High–risk/Low–risk	1.589	1.184–2.132	0.002
Age	> 65/<=65	2.123	1.101–4.092	0.025
Gender	Male/Female	0.841	0.393–1.801	0.656
Stage	II/I	1.362	0.663–2.799	0.401

**Figure 4:**
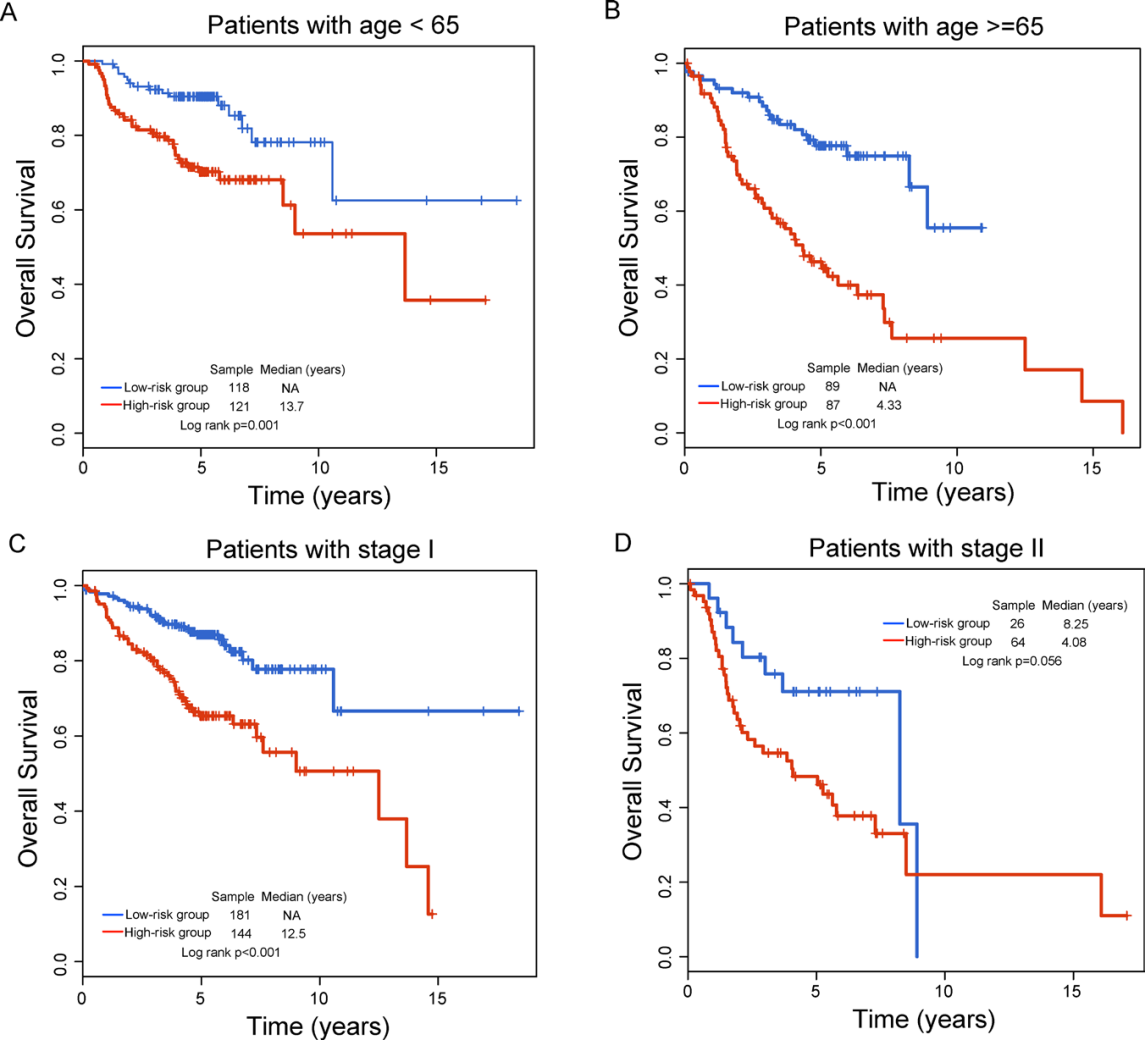
Stratification analysis of the seven-lncRNA signature for age and stage Kaplan-Meier survival curves between high-risk group and low-risk group for (**A**) young patients, (**B**) older patients, (**C**) stage I patients and (**D**) stage II patients.

Next, data stratification analysis was performed according to age or stage. First, all patients were stratified into young patients (< 65 years, *n* = 239) and older patients (> =65 years, *n* = 176). As shown in Figure 4A, the seven-lncRNA signature could subdivide younger patients into the high-risk group and low-risk group with significantly different survival time (*p* = 0.001, Figure 4A). For the older patients, the seven-lncRNA signature revealed the similar prognostic value (*p* < 0.001, Figure 4B). Then the seven-lncRNA signature was further tested for patients with the different stage. Patients with stage I (*n* = 325) in all datasets were also classified into two risk subgroups with significantly different survival time (*p* = 0.001, Figure 4C). For patients with stage II (*n* = 90), patients with high-risk score had obvious shorter survival than did those with low-risk scores (4.08 years vs. 8.25 years) despite the difference in survival is marginally significant (*p* = 0.056, Figure 4D). These results suggested that the prognostic value of the seven-lncRNA signature is independent of other clinicopathological factors for the survival of LUAD patients with early stage.

### Functional characteristics of the seven-lncRNA signature

To identify potential biological processes and pathways involved in the seven-lncRNA signature, we performed functional enrichment analysis for GO terms and KEGG pathways for protein-coding genes (PCGs) co-expressed with lncRNAs in the seven-lncRNA signature. For this purpose, we calculated the Pearson correlation coefficient between lncRNA and PCG using paired lncRNA and PCG expression profiles and chosen highly positively or negatively correlated PCGs (ranked top 0.5%) with at least one of seven lncRNAs. The functional enrichment analysis of GO and KEGG pathway revealed that PCGs correlated with lncRNAs clustered most significantly in three GO functional clusters (including cell cycle, chondrocyte differentiation and mRNA catabolic process) and four KEGG pathways (including cell cycle, ECM-receptor interaction, Focal adhesion and p53 signaling pathway) (Table 4).

**Table 4:** Significantly enriched functional clusters of GO terms and KEGG pathways

GO terms and KEGG pathways	NO. of genes	*P*-value	Fold Enrichment
**Functional clusters of GO terms**			
Cluster 1 (Enrichment Score: 1.95)			
GO:0051439~regulation of ubiquitin-protein ligase activity involved in mitotic cell cycle	7	0.002	5.104
GO:0042787~protein ubiquitination involved in ubiquitin-dependent protein catabolic process	20	0.002	2.192
GO:0051437~positive regulation of ubiquitin-protein ligase activity involved in regulation of mitotic cell cycle transition	11	0.014	2.427
GO:0031145~anaphase-promoting complex-dependent catabolic process	11	0.019	2.335
GO:0051436~negative regulation of ubiquitin-protein ligase activity involved in mitotic cell cycle	10	0.025	2.362
GO:0043161~proteasome-mediated ubiquitin-dependent protein catabolic process	18	0.092	1.502
Cluster 2 (Enrichment Score: 1.35)			
GO:0032331~negative regulation of chondrocyte differentiation	6	0.003	5.919
GO:0031641~regulation of myelination	4	0.066	4.193
Cluster 3 (Enrichment Score: 1.11)			
GO:0000184~nuclear-transcribed mRNA catabolic process, nonsense-mediated decay	16	0.006	2.218
GO:0006413~translational initiation	15	0.038	1.810
GO:0006614~SRP-dependentcotranslational protein targeting to membrane	11	0.059	1.922
GO:0019083~viral transcription	12	0.077	1.765
**KEGG pathway**			
hsa04110:Cell cycle	16	0.006	2.185
hsa04512:ECM-receptor interaction	12	0.013	2.336
hsa04510:Focal adhesion	20	0.034	1.644
hsa04115:p53 signaling pathway	23	0.043	2.022

## DISCUSSION

Lung adenocarcinoma is the most common form of non-small cell lung cancer. Early stage LUAD patients (stages I and II) were treated with surgical resection and adjuvant chemotherapy was required for LUAD patients with stage IB or stage II. Traditional clinical factors, including stage, tumor size, close margins and so on, were commonly used to guide treatment decisions for adjuvant chemotherapy. However, early stage LUAD patients still confronted the high risk of disease recurrence. A recent improvement on molecular mechanisms of LUAD has suggested that LUAD is a heterogeneous disease characterized by diverse morphologic and molecular features [27]. Molecular heterogeneous has been proven to be associated with the response to adjuvant chemotherapy [28]. Therefore, molecular markers are urgently needed to make further stratification for early-stage LUAD patients for identifying high-risk patients who will benefit from adjuvant chemotherapy and low-risk patients who will be able to avoid over-treatment. Significant efforts have been made to develop the molecular signature for predicting the risk of disease recurrence at the mRNA and miRNA levels [29–32].

In the past years, lncRNAs has gradually been elucidated as a key component of genome regulatory network. Alteration of lncRNA expression has been proven to be associated with cancer recurrence and metastasis [33]. However, genome-wide expression profiles of lncRNA are not widely available in most human cancers. Until now, several studies have reported a lncRNA-mining approach to obtain lncRNA expression profiles by repurposing the probes on the commonly used microarray platforms [20, 34–36], making it possible to look for lncRNA signature in diagnosis and prognosis prediction. More recently, some lncRNA signature associated with recurrence was identified in breast cancer [26, 37] and gastric cancer [38].

In this study, we obtained lncRNA expression profiles in a large number of early-stage LUAD patients by mining the existing microarray data on the Affymetrix HG-U133 Plus 2.0 array which is a widely used commercial platform. Then we examined the association between lncRNA expression and survival using univariate Cox regression model and identified 48 lncRNAs as candidate prognostic lncRNAs in the training dataset. However, so many prognostic lncRNAs are not conducive to clinical application. Moreover, the interrelated relationship among these candidate prognostic lncRNAs may exist. Therefore, we conducted multivariate Cox regression analysis for 48 lncRNAs and identified seven lncRNAs as independent biomarkers which greatly reduce the number of prognostic lncRNAs. These seven lncRNAs were integrated into a lncRNA-signature by risk score method based on their expression and relative contribution. By applying the seven-lncRNA signature to the training dataset, The signature was able to identify a high-risk subgroup and a low-risk subgroup of LUAD patients with significantly different survival (HR = 2.718, 95% CI = 2.054–3.597, *p* < 0.001). The majority of patients with the low signature score are likely to be survived longer than 6 years, whereas patients with high signature score likely survived less than 3.69 years (Figure 1). The prognostic value of the seven-signature was also verified in another two independent LUAD patient datasets, indicating good reproducibility of this seven-lncRNA signature in predicting the risk of survival for early-stage LUAD patients. More importantly, the association of the seven-lncRNA signature with survival was independent of other available clinicopathological factors including age, gender, stage and smoking status. Specifically, the seven-lncRNA signature was able to differentiate patients with poor survival and good survival within the same age or stage stratum, indicating the potential clinical utility of the seven-lncRNA signature in predicting the risk of recurrence for early-stage LUAD patients.

Although more and more lncRNAs were identified during the past years, functional study of lncRNA is still limited. Functional characteristics of seven lncRNAs have not been reported by our literature mining. Therefore, we performed bioinformatics analysis to identify correlated biological process and pathways by integrative analysis of lncRNA and PCGs. The results suggested that the seven-lncRNA signature may be involved in known lung cancer-related biological process and pathways. For example, it is well known that uncontrolled proliferation is one of hallmark of cancer, and many cell cycle regulators are altered in tumors [39, 40]. A recent study suggested that cell cycle-related biomarkers are important for prognosis prediction in LUAD [41]. The important function of ECM-receptor interaction and Focal adhesion in LUAD has been widely recognized [42, 43]. p53 signaling pathway genes have been found to be mutated in lung cancer. Moreover, p53 signaling pathway signaling pathway is significantly associated with the radio response of NSCLC [44].

In summary, we identified a novel lncRNA signature that was significantly associated with survival in patients with early-stage LUAD by probing and integrating currently available microarray data. Based on the seven-lncRNA signature, we constructed a risk score model which is able to classify patients into the high-risk group and the low-risk group with the significantly different clinical outcome. The robustness of the seven-lncRNA signature was successfully validated through application in the training dataset and other two independent patient datasets. Furthermore, the prognostic value of seven-lncRNA signature was independent of other clinicopathological factors. Our study highlighted the potential roles of lncRNAs as alternative molecular markers and therapeutic targets for early-stage LUAD patients.

## MATERIALS AND METHODS

### Patient datasets

LUAD patients with early-stage and their clinical data were downloaded from the GEO databases (http://www.ncbi.nlm.nih.gov/geo/). After removal of LUAD patients without survival status and early-stage, a total of 415 early-stage LUAD patients were analyzed in this study, including 127 patients from GSE50081 dataset (https://www.ncbi.nlm.nih.gov/geo/query/acc.cgi?acc = GSE50081) [45], 204 patients from GSE31210 dataset (https://www.ncbi.nlm.nih.gov/geo/query/acc.cgi?acc = GSE31210) [46] and 84 patients from GSE30219 dataset (https://www.ncbi.nlm.nih.gov/geo/query/acc.cgi?acc = GSE30219) [47]. The detailed clinical features of the early-stage LUAD patients were listed in Table 5.

**Table 5:** Clinical features of LUAD patients with early stage enrolled in this study

Covariates		GSE50081 *n* = 127	GSE31210 *n* = 204	GSE30219 *N* = 84
Age (years), no (%)	< 65	40 (31.5)	145 (71.1)	54 (64.3)
	> =65	87 (68.5)	59 (28.9)	30 (35.7)
Gender, no (%)	Male	65 (51.2)	95 (45.6)	65 (77.4)
	Female	62 (48.8)	109 (53.4)	19 (22.6)
Vital status, no (%)	Alive	88 (72.7)	174 (98.1)	191 (84.5)
	Dead	76 (27.3)	30 (1.90)	39 (15.5)
Tumor stage, no (%)	I	92 (72.4)	162 (79.4)	71 (84.5)
	II	35 (27.6)	42 (20.6)	13 (15.5)
Smoking status, no (%)	Never-smoker	23 (18.1)	105 (51.5)	−
	Ever-smoker	92 (72.4)	99 (48.5)	−
	Undetermined	9.5		−

“−” means no corresponding information available in the dataset.

### Acquisition and processing of lncRNA expression profiles

Original raw microarray data of three patient datasets profiled on the Affymetrix HG-U133 Plus 2.0 platform were downloaded from the GEO databases. Each microarray dataset was processed and normalized using the Robust Multichip Average (RMA) algorithm including background correction, quantile normalization and log2-transformation [48]. The Z-score transformation was performed independently in each dataset in order to account for differences in systematic measurement among multiple microarray datasets [49].

lncRNA expression profiles of LUAD patients were obtained by repurposing the microarray probes as previously described [25, 34] as follows: (i) The probe set sequences of Affymetrix HG-U133 Plus 2.0 were re-mapped to the human genome (GRCh38) using SeqMap software [50]. (ii) Those probes that were uniquely mapped to the human genome with no mismatch were kept. (iii) The chromosomal position of the remaining probes was matched to the chromosomal position of lncRNA genes from GENCODE (release 23). Finally, a total of 3578 lncRNA-specific probes and 2332 corresponding lncRNAs were obtained. The expression value of lncRNAs with multiple probes was calculated by using the mean value of multiple probes.

### Statistical analysis

The univariate Cox regression model was used to evaluate the association and to identify candidate prognostic lncRNAs that were significantly associated with survival at adjusted *p*-value < 0.05 after Bonferroni correction. Considering that there was an interrelated relationship among candidate prognostic lncRNAs, multivariate Cox regression analysis was used to identify independent lncRNA biomarkers in predicting the risk of recurrence. A risk score model was built by including expression values of each independent lncRNA biomarkers, weighted by their estimated regression coefficients in the multivariate Cox regression analysis as follows: Risk Score = (0.0912*FLJ30679)+(0.2493*LINC00511) +(-0.3009*CTC-429P9.1)+(0.5554*LINC01127)+(0.1233 *MIF-AS1)+(0.2251*RP11-278J6.4)+(−0.5957*RP11-25K19.1). The Kaplan-Meier survival curves were used to compare the difference in survival between high-risk group and low-risk group. The statistical significance was examined by log-rank test. Univariate and multivariate analyses with Cox proportional hazards regression for survival were conducted for individual clinical factors with the lncRNA signature in each dataset. Hazard ratios (HR) and 95% confidence intervals (CI) were calculated. The prognostic performance at five years was accessed using time-dependent receiver operating characteristic (ROC) curves. All statistical analyses were conducted using R software and Bioconductor.

### Functional enrichment analysis

Biological processes or pathways involved in lncRNA signature were predicted using functional enrichment analysis of GO and KEGG in DAVID Bioinformatics Resources 6.8 (https://david.ncifcrf.gov/) [51, 52]. Significant GO terms and KEGG pathway with *p* < 0.05 were identified limited in “GOTERM_BP_DIRECT” and “KEGG_PATHWAY” with the reference human genome as background.

## SUPPLEMENTARY MATERIALS TABLE




