# Supplementary material for: A novel seven-long non-coding RNA signature predicts survival in early stage lung adenocarcinoma
Source: Oncotarget. 2017 Jan 21;8(9):14876–86. doi: 10.18632/oncotarget.14781 (PMC5362451; doi:10.18632/oncotarget.14781)
Supplement: Supplementary file 1 [file oncotarget-08-14876-s001.pdf]

A novel seven-long non-coding RNA signature predicts survival in early stage lung adenocarcinoma

Supplementary Materials

Supplementary Table 1: Detailed information of 48 lncRNAs that are significantly associated with survival from the training dataset (adjusted *p*-value < 0.05 after Bonferroni correction). See Supplementary_Table_1
